# Effect of Exposure to Blue Light from Electronic Devices and the Mediterranean Diet on Macular Pigment

**DOI:** 10.3390/jcm13247688

**Published:** 2024-12-17

**Authors:** Marta-C. García-Romera, Víctor Ponce-García, Úrsula Torres-Parejo, Alfredo López-Muñoz

**Affiliations:** 1 Department Condensed Matter Physics, Optics Area, Vision Research Group (CIVIUS), University of Seville, 41004 Sevil, Spain; vicpongar@alum.us.es (V.P.-G.); alopez57@us.es (A.L.-M.); 2 Department of Statistics and Operations Research, University of Granada, 18071 Granada, Spain; ursula@ugr.es

**Keywords:** macular pigment optical density, blue light exposure, exposure to LEDs, Mediterranean diet, carotenoids

## Abstract

**Objective:** To explore the effect of time exposure to flat screen electronic devices with LED lighting and the Mediterranean diet on macular pigment optical density (MPOD). **Methods:** In this cross-sectional observational study, the MPOD was measured by heterochromatic flicker photometry in 164 eyes (47 of younger women aged 20–31 and 35 of older women aged 42–70). Exclusion criteria: evidence of macular degeneration and eyes with cataracts. Data on the use of electronic devices and Mediterranean diet adherence were collected through a survey. Nonparametric analysis of variance and independent sample *t*-tests were used to compare subjects. **Results:** Significant differences (*p* < 0.01) were found in total time of exposure to LEDs (hours per day) between both groups (9.31 ± 3.74 younger women vs. 6.33 ± 3.64 older women). The MPOD values for the younger and adult populations were significantly different: 0.38 ± 0.16 and 0.47 ± 0.15 (*p* < 0.01), respectively. When comparing both groups for the same time of exposure to LEDs, differences were obtained between MPOD values of both populations: For total exposures greater than 6 h per day, the MPOD values were lower in younger women than in adult ones (0.37 ± 0.14 vs. 0.50 ± 0.14, *p* < 0.01). On the other hand, a significantly higher adherence was found in the older women in comparison with the younger women (OW 9.23 ± 2.50 vs. YW 7.70 ± 2.08, *p* < 0.01), with higher MPOD values (OW (0.52 ± 0.14) vs. (YW (0.34 ± 0.18). **Conclusions:** Higher MPOD values are observed with decreasing exposure time to electronic devices with LED lighting screens and higher adherence to the Mediterranean diet.

## 1. Introduction

The development of illumination systems based on light-emitting diodes (LEDs) is spread worldwide. Today, more than 90% of all light sources are SSL (solid state lighting), LED technology, with different display configurations of light-emitting diodes (OLED, AMOLED, etc.) LEDs are more energy-efficient and have a smaller size, which makes them suitable for any small electronic device, such as cell phones, tablets, and for flat-screen televisions and computer monitors. The spectral emission of LEDS is mainly white light, including a proportion of light at wavelengths of 435 to 440 nm, which are included in the ‘blue light hazard’ (BLH) assessment. BLH is defined as the photochemical risk to the retinal tissues of the eye or photomaculopathy, which occurs when looking at bright sources, such as the sun [1]. Although the BLH irradiances that are emitted by electronic devices with visual displays are far from those emitted by the sun [2], the extensive usage of electronic devices (time of exposure) is a matter of growing public concern because we are gradually being exposed to more sources of blue light and for longer periods of time [3].

The negative effects of the extended usage of these devices, such as computer vision syndrome (CVS) [4], are becoming a major public health issue with eye strain symptoms that could be caused by poor refractive compensation [5] and/or excessive time of exposure (TE) [6]. CVS is influenced by the observation distance or angle and by a decrease in the quality or frequency of blinking [7]. Blue light also plays a key role in physiological functions, such as in the regulation of circadian rhythms of sleep and wakefulness [8]. The relationship between TE to SSL screen displays (380–780 nm) [6] and circadian rhythm disorders has also been explored [8,9]. In young human eyes, it has been reported that short-term exposure to blue light resulted in inhibition of axial elongation, which may have an impact on myopia control, which needs to be explored [10]. Blue light has long been known to be phototoxic to the retina [11]. Several studies have been conducted in retinal cell cultures and mice exposed to LED illumination to investigate the effect of SSL screens on retinal damage. The negative effect of visual blue light is due to a decrease in mitochondrial function associated with retinal ganglion cells and photo oxidative damage of photoreceptor cells, leading to the appearance of reactive oxygen species with cellular apoptosis [12].

The main two carotenoids, lutein (L) and zeaxanthin (Z), found in the retina and known as macular pigment (MP), are selectively concentrated in the macula lutea (or central 1.5 mm of the macula) and the levels decrease exponentially to a concentration of zero with 6–8 degrees of eccentricity [13]. MP density is a measurement of the level of these xanthophylls in the retina [14] that can be measured by different techniques ‘in vivo’, with heterochromatic flicker photometry (HFP) being one of the most used methods among the psychophysical techniques [15]. MP is considered to protect the retina by filtering blue light [16] and has recently been related with visual quality [17], while low levels are associated with macular degeneration [18]. The macular carotenoids L and Z cannot be synthesized by humans, and fruits and vegetables are the main dietary sources [16]. The Mediterranean diet (MD) is characterized by a high consumption of fruits and vegetables and its protective role on the progression of age-related macular diseases has been evaluated by the Mediterranean diet score, showing that a higher adherence may play a protective role against age-related macular degeneration [19]. Besides diet, other internal and external factors have been investigated in relation to MPOD, such as age [20], sex and body mass index or fat percentage [21], iris color or ethnicity [22], and also other lifestyle factors such as smoking, alcohol consumption, exercise [17], and time of exposure to ultraviolet light [8]; however, correlation with time exposure to blue light from electronic devices has been scarcely investigated so far.

We hypothesized that a longer exposure time to electronic devices and a lower adherence to the Mediterranean diet could be related to a lower density in the MP evaluation as optical density. The aim of this work was to collect information about time of exposure to electronic devices based on LED technology (LED/OLED/AMOLED) and adherence to the Mediterranean diet in a group of healthy women of different ages and explore the relationship with MP density evaluated by HFP.

## 2. Materials and Method

### 2.1. Study Design and Population

The study protocol (DENPIGMA-0831-N-17-f) was approved by the local ethics committee (“Comité Coordinador de Ética de la Investigación Biomédica de Andalucía”), and the experimental procedures adhered to the Declaration of Helsinki. Eighty-Six women were enrolled in a cross-sectional observational study conducted in the Optometry Clinic of the Faculty of Pharmacy of the University of Seville between October, 2021 and February, 2022. This study was advertised for women over 18 years of age among students, faculty personnel, and patients who visited the Optometry Clinic.

To reduce the variability related to race, gender, or some systemic illnesses, only healthy, Caucasian, non-smoking women were recruited. Exclusion criteria included evidence of AMD, evaluation with an Amsler grid [23] or history of hereditary AMD, or lens opacity (cataract) explored with a slit lamp. All recruited participants were aware of the experimental hypothesis, and informed consent was obtained before starting the experiment.

The participants were divided into two groups according to their age considering that the use of electronic devices started in Spain in the 1990s. The younger women group (YW) included women 20 to 31 years of age, and the older women group (OW) included women 42 to 70 years of age. Height and weight were also recorded to calculate the body mass index (BMI), which was classified according to OMS [24] as underweight, <18.5 kg/m^2^; normal weight, ≥18.5–24.9 kg/m^2^; overweight, 25.0–29.9 kg/m^2^; and obese, 30.0 kg/m^2^.

### 2.2. Macular Pigment Optical Density (MPOD) Evaluation

MPOD was measured using HFP [25,26] with an MPS II (Macular Pigment Screener II) by Elektron Technology UK Ltd. (Cambridge, UK).

MP absorbs selectively in the blue region of the visible spectrum, at 460 nm, and it is present only in the central eight degrees of the macula. HFP is performed for central fixation where MP is maximal. The MPS II uses low-intensity light of two specific wavelengths (460 nm and 540 nm) at calibrated intensities to gauge a patient’s heterochromatic flicker response. Two measurements are taken, one with the patient looking directly at the stimulus target (using the central region of the macula, 0.25°–0.5° of eccentricity) (absolute MPOD values) and another where the patient fixates peripherally on a point 8° to the side of the stimulus light (so it is viewing the stimulus where MP density is minimum) [27]. Both measurements lead to estimated MPOD values. The MPOD was measured in density units (du) and ranged from 0 to 1.

### 2.3. Screen Time Usage During Daytime

All participants completed a structured questionnaire with open-ended questions. They were asked about the electronic devices they usually use (such as television, tablet, PC, laptop, eBook, or mobile phone) and the average time participants spent on screen-based activities per day. In addition, they were asked about the brand and model of each device. This questionnaire was completed at home, and the answers were checked at the delivery. The light sources of the electronic devices were then searched for in the technical datasheet of the instruments or obtained from the companies when necessary.

According to the daily time of exposure (TE) to LEDs, two main groups were considered, those with 0–6 h/daily versus those with > 6 h, considering that during working hours, PC and computer screens are used, and mobile phones are consulted. This classification was made considering a distribution of 24 h a day that allows for sleeping around 8 h, working another 8, and spending the remaining 8 eating and doing leisure activities.

### 2.4. Adherence to the Mediterranean Diet: The Mediterranean Diet Score (MES)

The volunteers completed a validated questionnaire consisting of 14 questions to evaluate the adherence to the Mediterranean Diet [28]. The score ranged from 0 to 14 as a result of evaluating with 1 point the consumption of key food items, such as olive oil as the main fat fruit, vegetables or salad, wine, fish, legumes or nuts, preferentially consumption of white meat vs. red meat, consumption of sauté, and low consumption of red meat or meat products. We applied the following criteria for categorization of the adherence to the MD: weak adherence, ≤ 5; moderate to fair adherence, 6–9; good or very good adherence ≥ 10 [28].

### 2.5. Statistical Analyses

A Shapiro–Wilk test and Kolmogorov–Smirnov test were used for normality. For the normally distributed data, independent sample *t*-tests were used to make comparisons between population subgroups. *p* < 0.05 was considered statistically significant. Nonparametric analysis of variance using a Kruskal–Wallis test and a Mann–Whitney U test were conducted. All statistical analyses were performed with StatGraphics Centurion Plus 5.1 (Statgraphics Technologies, Inc., The Plains, VA, USA) and SPSS v.23 (IBM Corp., Armonk, NY, USA).

## 3. Results

Eighty-Six women were initially enrolled in the study, but only eighty-two completed all the requested data and met the inclusion criteria. Table 1 shows the descriptive statistics and MPOD values in the group of volunteers participating in the study and by age group.

Absolute and estimated MPOD values were measured in both eyes of the participants. Due to a certain degree of difficulty in concluding the measurement with the technique of HFP employed by the MPS II instrument, only 158 eyes completed the evaluation of MPOD (absolute and estimated value). The mean absolute and estimated MPOD values that were measurable were similar (RE 0.41 ± 0.16 and LE 0.42 ± 0.16 and RE 0.43 ± 0.16 and LE 0.43 ± 0.16, respectively) with no significant difference; therefore, the measures were randomized from both eyes.

The inter-ocular correlation coefficient between subjects’ fellow eyes was 0.63 (*p* < 0.01) for absolute values and 0.72 (*p* < 0.01) for estimated values. Absolute values for each participant were considered for further statistical treatments.

The mean age of the participants was 36.8 ± 16.2 (range 20–70 years). The younger women (YW) group (*n* = 47) included women from 20 to 31 years old and mean age of 23.6 ± 2.2. The older women (OW) group (*n* = 35) included women from 42 to 70 with a mean age of 54.7 ± 7.5. The YW group showed significantly lower values for MPOD than the older women (OW) group: 0.38 ± 0.15 (range 0.12–0.70) vs. 0.47 ± 0.16 (range 0.17–0.89) (*p* < 0.01), respectively.

The mean BMI of the participants was 22.9 ± 3.9 (range 16.3–35.4). YW and OW BMI were significantly different (21.9 ± 3.5 YW vs. 24.1 ± 4.2 OW) (*p* ≤ 0.01). A higher prevalence of excess weight in the OW group (23.8% vs. 13%) was observed. There was no significant difference in MPOD among the different BMI categories (Table 1).

### 3.1. Light Sources of the Electronic Devices and Time of Exposure by Age Groups

**Figure 1:**
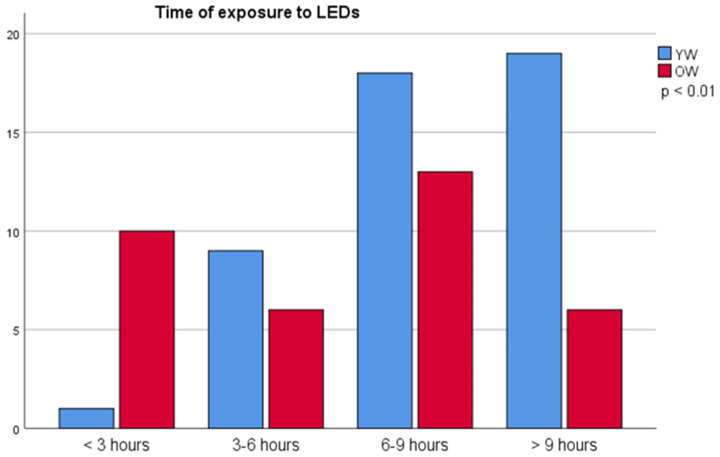
Time of exposure to SSL screens vs. population (YW: younger women, OW: older women).

To assess the TE to LEDs, the different devices reported by the volunteers were investigated for the source of light in the instruction manuals. In tablets, laptops, and PCs, 100% of the light sources were based on LEDs, while in mobile phones, 82.10% were LED, and 17.09% were OLED/AMOLED. In the televisions (TVs), the vast majority of the reported devices had LED as light source (64.9%), 24.6% (flat TVs without LED) had cold cathode fluorescent lamp, only 7% had cathode ray tube, and 3.5% had plasma display panel. The total time of exposure (TE) to different devices by age groups is shown in Figure 1. It can be observed that younger women have a higher use of LEDs than the older ones (TE > 9 h/day: 40.4% YW vs. 17.6% OW) and (TE < 3 h/day: 2.1% YW vs. 28.6% OW).

A significant difference in the TE to all kinds of screens between the two age groups was observed, with YW having a significantly higher TE (*p* < 0.01) to LEDs than the OW group (9.31 ± 3.74 vs. 6.33 ± 3.64).

The devices reported in this study are shown in Table 2, together with the TE by age group. Among all electronic devices, computers (laptop plus PC) were had the highest time of usage, with a mean of 3.57 ± 2.82 h/day, followed by mobile phones (3.29 ± 2.41 h/day), TVs (2.05 ± 1.32), and tablets and e-books (0.58 ± 1.05 and 0.01 ± 0.11 h/day, respectively). In the younger women (YW) group, the most widely used device was the mobile phone, while personal computers were the most popular in the older women (OW) group.

### 3.2. Time of Exposure to LEDs and MPOD

Out of the 158 eyes studied for MPOD, 94 belong to YW and 64 to OW. The mean value for (YW + OW) MPOD was 0.42 ± 0.16 (range 0.02–0.89).

When the sample was grouped by age, a significant difference in MPOD values was observed. The YW group showed significantly lower values than the older group: 0.38 ± 0.16 (range 0.02–0.74) and 0.47 ± 0.15 (range 0.17–0.89) (*p* < 0.01).

**Figure 2:**
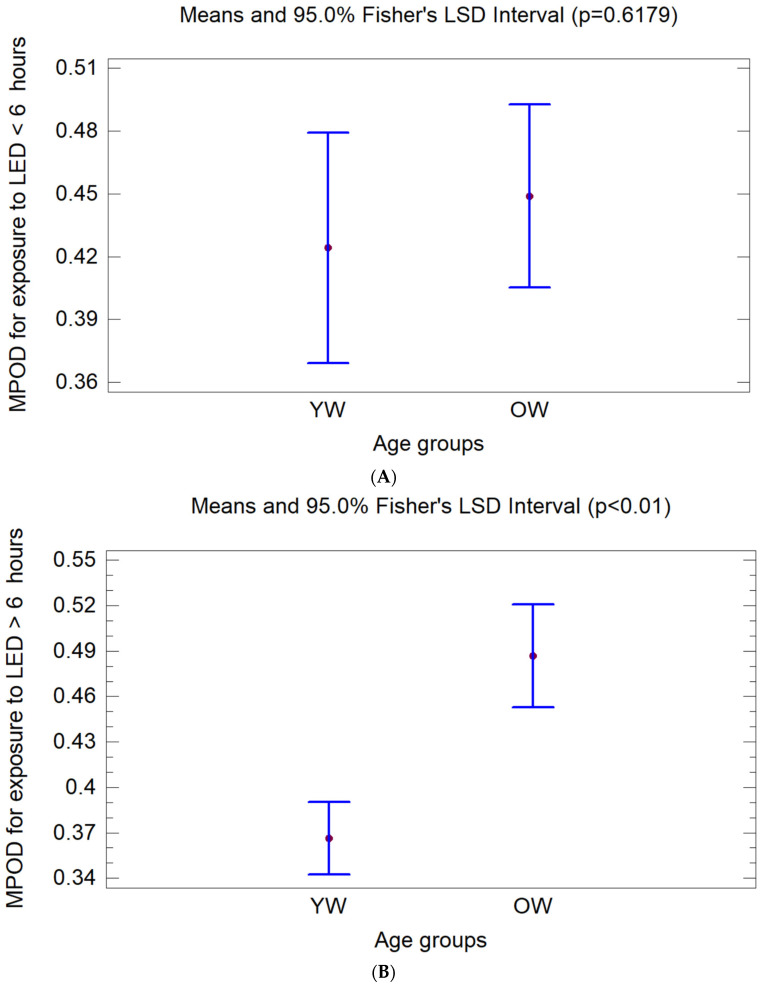
(**A**,**B**): Macular pigment optical density (MPOD) (mean ± 95% confidence interval) in relation to time of exposure to SSL screens (hours/day), grouped by age. (YW: younger women, OW: older women).

Considering exposure to LEDs >6 h/day, a significant difference in MPOD values was observed in age groups: (YW 0.37 ± 0.14 vs. OW 0.50 ± 0.14) (*p* < 0.01) (Table 1) (Figure 2A).

No significant differences in MPOD values are found between younger and adult women when the exposure time is less than 6 h (Figure 2B).

### 3.3. Mediterranean Diet

In relation to the adherence to the Mediterranean diet (MD) (Table 1), a significantly higher adherence was found in the OW group in comparison with the YW (YW 7.70 ± 2.08 vs. OW 9.23 ± 2.50, *p* < 0.01), with 48.6% of participants with very good adherence in the OW group vs. only 19.1% in YW group. All the participants used olive oil as the main dietary fat, and 71% reported consuming more than four spoons a day. This proportion was even higher (80%) among the OW. MPOD was also significantly higher in the women with good or particularly good adherence compared to those with moderate adherence in the total sample. The same trend towards increasing MPOD with increasing adherence to the MD was also observed in YW, although in this case, these differences did not reach a significant level. From the 14-item tool of adherence to the Mediterranean Diet, we analyzed in detail the two questions related to consumption of fruits, vegetables and fish. 38% of the participants referred consuming three or more units of fruit per day and 56% two servings or more of vegetables a day. The OW group was over this value compared to the YW group. A similar trend was observed with fish consumption, revealing the better dietary habits in the OW group compared to the YW. A higher value for MPOD was found in the total sample for those consuming three or more units of fruit per day.

MPOD values were studied, considering groups by age and high–low adherence to the Mediterranean diet, 24 YW (0.34 ± 0.18)–23 YW (0.40 ± 0.16), and 18 OW (0.52 ± 0.14)–17 OW (0.41 ± 0.11).

In addition, MPOD values were analyzed according to consuming or not three or more pieces of fruit: (14 YW (0.44 ± 0.12)–33 YW (0.34 ± 0.18), *p* < 0.05,)–(17 OW (0.48 ± 0.17)–18 OW (0.45 ± 0.10), *p* > 0.05,). Furthermore, there were differences in the MPOD values between YW and OW whose intakes of fruit were lower than three pieces per day ((0.34 ± 0.18 YW)–(0.46 ± 0.14 OW), *p* < 0.05).

In addition, assuming the positive effect of fruits and vegetables, both rich in L and Z, the intake of three or more pieces of fruit plus two servings/day of vegetables or salad was considered. Differences are observed among YW and OW (*p* < 0.05) when fruits or vegetables are consumed. In addition, the percentage consumption of fruits and vegetables is different (*p* < 0.05) among age groups (Table 1).

## 4. Discussion

We evaluated the adherence to the Mediterranean diet and time of exposure to LEDs light in a group of healthy women to explore its connection with MPOD measured by HFP. Also, other factors, such as age and body mass index, were analyzed in the studied group.

The absolute MPOD mean value for the total sample (0.42 ± 0.16; range 0.12–0.89) was higher than previously published values for Spanish women measured with the same methodology and in a similar age interval (20–65 years) 0.342 ± 0.155 [29], although the authors do not indicate if that value corresponded to absolute or estimated measures. To obtain the absolute value both, foveal and para-foveal data must be obtained, which is a time-consuming process, and for that reason, most studies use the more simplified value, namely the estimated value of MPOD from only the foveal data calculated with the manufacture’s empirical algorithm, which considers the subject’s age [30]. Previous studies have shown that estimated values in healthy aged populations are lower than absolute values; however, absolute and estimated values were not different [31], in accordance with our findings. The values for MPOD obtained in the population included in this study are consistent with recent published data including Caucasian women [32].

MPOD increased significantly with age, with OW having a significantly higher MPOD values than YW. This is a striking result, since an inverse association between MPOD and age has been established in different populations [33,34]. However, the relationship between age and MPOD is not straightforward. For instance, Pipis et al. [35], in a Central European population using fundus reflectometry, reported an increase in MPOD with age, along with a more eccentric distribution of MP profiles. Lima et al. [20] using a dual-wavelength autofluorescence method in healthy subjects, reported that MPOD values were highest near the foveal center and that those values increased during adulthood (peak at 45–50 years), followed by a gradual reduction after 60 years of age [36]. Berendschot and Van Norren compared five different methods to measure MPOD, including fundus reflectance spectroscopy and HFP, and reported significant (*p* < 0.001) bivariate correlation coefficients among them. After exploring the association of MPOD with age using these techniques, they concluded that there was no age effect in the MPOD. Only MPOD values obtained with HFP showed a small, but significant, decrease with age that could be caused by an increase in the parafoveal data, suggesting that the central MPOD is unchanged with age [37].

In our study, we also found a significant positive association between MPOD and BMI for the total sample. Hammond et al. [38] established an inverse relationship between MPOD and BMI (*n* = 680, r = −0.12, *p* < 0.0008) and percentage of body fat, assessed by bioelectric impedance (*n* = 400, r = −0.12, *p* < 0.01). A competitive uptake of MP into the body fat and the retinal tissue was postulated by Johnson et al. [39]. However, Ji et al. [40], in a sample of 281 healthy Chinese individuals, including 96 males and 185 females with ages ranging from 17 to 85 years, reported no statistically significant association of MPOD with BMI. In the study by Hammond et al. [38], the higher body fat percentage was related to lower serum carotenoid levels and to lower dietary carotenoid intake. Thus, a factor to consider is diet, since it is known that a diet rich in the macular carotenoids L and Z favors the increase in MPOD [15]. In a recent study on a Spanish population, Olmedilla-Alonso et al. [29] reported that MPOD shows age-specific correlations with fruit and vegetable intake, with intake being higher in older subjects compared to younger ones. This is in accordance with our results related to the Mediterranean diet score and fruit consumption. MPOD values were significantly higher in women with higher adherence (with 10 points or over) to the Mediterranean diet, which were 48.6% in the OW vs. only 19.1% of the YW. The volunteers included in this study were all from the south of Spain (Seville and surroundings), and this area is very influenced by the Mediterranean diet, characterized, among other foods, by the consumption of fruits and vegetables. Estevez-Santiago et al. [41] identified vegetables and green foods of plant origin as the major contributors to L + Z intake in the Spanish diet, while red/orange foods and fruits showed the strongest relationship to MPOD in the participants of 45–65 years of age. In accordance with our findings, higher (*p* < 0.05) MPOD values were found in the participants reporting intake of three or more units of fruit a day, which were 51.4% in the OW group vs. only 29.8% in the YW group.

Another factor to be considered is the time of exposure to LEDs. YW reported a higher (*p* < 0.01) exposure to electronic devices, which probably is sustained over time, compared to that of adult women, since YW (23.6 ± 2.2 years) have developed their activity from early ages exposed to electronic devices, while for OW (54.5 ± 7.5 years), they not only reported lower TE to electronic devices, but also the use of them corresponds only to the last period of their lives. Few studies have evaluated the spectral emission of electronic devices and compared them to exposure conditions with international exposure limits and with the exposure likely to be received from staring at a blue sky. None of the sources assessed approached the exposure limits, even for extended viewing times. However, in other recent investigations in rodents [42], blue light exposure (LEDs, 450–500 lux, 400 to 490 nm) during 28 days on a 12:12 h light–dark cycle induced a significant hazard to the visual system, resulting in damage to the retina with the associated remodeling of visual cortex neurons. Similarly, in human adult retinal epithelial cells [43] illuminated using display devices with different blue light wavelength ranges, 449 nm, 458 nm, and 470 nm, they reported an increased production of reactive oxygen species (ROS) and apoptosis in retinal cells for the display with a blue light peak at a shorter wavelength.

Therefore, in this population, three important facts can be highlighted: First, there is an increase in macular pigment optical density (MPOD) with age, which can be explained by a greater adherence to the Mediterranean diet, characterized by higher fruit consumption in the older population. This is supported by numerous studies linking increased MPOD with the consumption of lutein (L) and zeaxanthin (Z). Second, there is a lower use of electronic devices in the older population, a finding supported by scientific studies conducted on rodents exposed to extreme conditions.

## 5. Conclusions

The main conclusion could be that the most important factor in MPOD level is a diet rich in L and Z, rather than age or the use of electronic devices.

In addition, it could be considered that almost all electronic devices are based on LEDs technology, and YW are heavier users than older ones. The YW group showed a tendency to decrease the MPOD with increasing time exposure to electronic devices with SSL lighting screens, although no statistically significant differences were obtained. Considering over 6 daily hours of exposure, there were significant differences in MPOD values between the populations of younger and adult women, with higher MPOD values for the OW group. Finally, MPOD values were significantly higher in women with higher adherence to the Mediterranean diet. MPOD values are higher when a representative amount of fruit is consumed. The OW group had better dietary habits compared to the YW.

## Figures and Tables

**Table 1:** Analysis of the macular pigment optical density (MPOD) values considering population characteristics, such as age, body mass index (BMI), time of exposure to LEDs devices, Mediterranean diet, or dietary background.

	Factors (Mean ± sd)			Absolute MPOD (Mean ± sd)			Estimated MPOD (Mean ± sd)		
	Total Sample (*n* = 82)	Younger Women (YW) (*n* = 47)	Older Women (OW) (*n* = 35)	Total Sample	YW	OW	Total Sample	YW	OW
Age (*y*)	36.8 ± 16.2	23.6 ± 2.2 ^a^	54.5 ± 7.5 ^b^	0.42 ± 0.16	0.38 ± 0.15 ^a^	0.47 ± 0.16 ^b^	0.43 ± 0.16	0.39 ± 0.15 ^a^	0.47 ± 0.15 ^b^
*Range* (*min–max*)	20–70	20–31	42–70	(0.12–0.89)	(0.12–0.70)	(0.17–0.89)	(0.10–0.86)	(0.12–0.86)	(0.10–0.72)
BMI (kg/m^2^)	22.9 ± 3.9	21.9 ± 3.5 ^a^	24.1 ± 4.2 ^b^						
*Range* (*min–max*)	16.3–35.4	16.7–34.3	16.3–35.4						
BMI distribution (*n* (%))									
Underweight	7 (8.5%)	4 (8.7%)	3 (8.8%)	0.39 ± 0.16	0.31 ± 0.19	0.48 ± 0.02	0.43 ± 0.14	0.37 ± 0.16	0.51 ± 0.06
Normal weight	54 (65.9%)	36 (78.3%)	18 (52.9%)	0.40 ± 0.17	0.38 ± 0.15	0.46 ± 0.20	0.43 ± 0.16	0.40 ± 0.16	0.49 ± 0.16
Weight excess (overweight + obesity)	19 (23.2%)	6 (13.0%)	13 (23.8%)	0.48 ± 0.11	0.46 ± 0.09	0.49 ± 0.11	0.43 ± 0.15	0.41 ± 0.16	0.43 ± 0.16
Time of exposure hour/day (h/d) to Leds	8.0 ± 4.0	9.3 ± 3.7 ^a^	6.3 ± 3.6 ^b^						
<6 h/d	26 (31.7%)	10 (21.3%)	16 (45.7%)	0.45 ± 0.17	0.46 ± 0.15	0.45 ± 0.18	0.43 ± 0.17	0.44 ± 0.15	0.43 ± 0.18
>6 h/d	56 (68.3%)	37 (78.7%)	19 (54.3%)	0.41 ± 0.15	0.37 ± 0.14 ^a^	0.50 ± 0.14 ^b^	0.42 ± 0.15	0.38 ± 0.16 ^a^	0.50 ± 0.12 ^b^
Mediterranean diet score (14 points)	8.4 ± 2.4	7.7 ± 2.1 ^a^	9.2 ± 2.5 ^b^						
*Range* (*min–max*)	1–13	2–11	1–13						
weak adherence ≤ 5 *n*, (%)	9 (11.0%)	7 (14.9%)	2 (5.7%)	0.45 ± 0.181.2	0.43 ± 0.18	0.53 ± 0.24	0.46 ± 0.15	0.42 ± 0.15	0.57 ± 0.06
moderate to fair adherence 6–9, *n* (%)	47 (57.3%)	31 (66.0%)	16 (45.7%)	0.38 ± 0.151	0.36 ± 0.15	0.43 ± 0.15	0.39 ± 0.16	0.37 ± 0.16	0.42 ± 0.18
good or very good adherence ≥10, *n* (%)	26 (31.7%)	9 (19.1%)	17 (48.6%)	0.49 ± 0.142	0.44 ± 0.10	0.48 ± 0.16	0.47 ± 0.13	0.44 ± 0.14	0.49 ± 0.12
Dietary background (rations per day or week)									
Olive oil (≥4 d)									
YES	58 (70.7%)	30 (63.8%)	28 (80.0%)	0.42 ± 0.16	0.37 ± 0.14	0.48 ± 0.16	0.43 ± 0.15	0.37 ± 0.15	0.48 ± 0.15
NO	24 (29.3%)	17 (36.2%)	7 (20.0%)	0.42 ± 0.15	0.40 ± 0.15	0.47 ± 0.16	0.42 ± 0.16	0.44 ± 0.15	0.43 ± 0.16
Fruit (≥3 d)									
YES	31 (37.8%)	14 (29.8%)	18 (51.4%)	0.46 ± 0.141	0.44 ± 0.121	0.48 ± 0.15	0.44 ± 0.13	0.43 ± 0.11	0.45 ± 0.14
NO	51 (62.2%)	33 (70.2%)	17 (48.6%)	0.38 ± 0.172	0.34 ± 0.182 ^a^	0.46 ± 0.14 ^b^	0.41 ± 0.17	0.38 ± 0.17 ^a^	0.48 ± 0.16 ^b^
Vegetable (≥2 d)									
YES	46 (56.1%)	23 (48.9%)	23 (65.7%)	0.44 ± 0.15	0.39 ± 0.12	0.48 ± 0.16	0.43 ± 0.13	0.40 ± 0.13	0.47 ± 0.13
NO	36 (43.9%)	24 (51.1%)	12 (34.3%)	0.40 ± 0.17	0.37 ± 0.17	0.46 ± 0.16	0.41 ± 0.18	0.39 ± 0.18	0.46 ± 0.18
Fruit (≥3 d) and vegetable (≥2 d)									
YES	23 (28%)	8 (17%) ^a^	15 (42.9%) ^b^	0.46 ± 0.15	0.45 ± 0.13	0.47 ± 0.17	0.45 ± 0.17	0.46 ± 0.15	0.45 ± 0.18
One of two things	31 (37.8%)	21 (44.7%) ^a^	10 (28.6%) ^b^	0.38 ± 0.17	0.33 ± 0.18 ^a^	0.48 ± 0.10 ^b^	0.42 ± 0.17	0.37 ± 0.15 ^a^	0.53 ± 0.16 ^b^
NO	28 (34.1%)	18 (38.3%) ^a^	10 (28.6%) ^b^	0.41 ± 0.15	0.39 ± 0.16	0.44 ± 0.13	0.42 ± 0.14	0.41 ± 0.15	0.45 ± 0.15
Fish consumption (≥3 w)									
YES	28 (34.1%)	13 (27.7%)	15 (42.9%)	0.44 ± 0.15	0.40 ± 0.12	0.46 ± 0.14	0.46 ± 0.16	0.40 ± 0.13	0.45 ± 0.13
NO	54 (65.9%)	34 (72.3%)	20 (57.1%)	0.42 ± 0.16	0.37 ± 0.17	0.49 ± 0.18	0.41 ± 0.15	0.39 ± 0.18	0.48 ± 0.16

For each variable, different letters indicate significant differences (*p* < 0.05) between YW and OW columns, and different numbers indicate significant difference among rows in the same column for each variable.

**Table 2:** Time of exposure (mean ± standard deviation) in hours/day for different devices containing LEDs as light sources for the two groups of women: the younger group (YW) and the older group (OW).

Device	YW	OW
Mobile phone	4.60 ± 2.28	1.57 ± 1.17 **
Laptop	2.73 ± 2.32	1.08 ± 1.60 **
TV	1.95 ± 1.24	2.20 ± 1.44
Tablet	0.76 ± 1.21	0.31 ± 0.69
PC	0.59 ± 1.67	3.00 ± 3.23 *
Total LEDs	9.31 ± 3.74	6.33 ± 3.64 *

* (*p* < 0.05) ** (*p* < 0.01).

## Data Availability

The raw data supporting the conclusions of this article will be made available by the authors on request.

## Glossary

L	Lutein
Z	Zeaxanthin
BLH	blue light hazard
MP	macular pigment
MPOD	macular pigment optical density
LEDs	light emitting diodes
CVS	computer vision syndrome
AMD	age-related macular degeneration
YW	younger women group
OW	older women group
HFP	heterochromatic flicker photometer
MPS II	Macular Pigment Screener II
TTE	Total time of Exposure
